# Knowledge Deficits Among Community Pharmacists May Drive Antibiotic Resistance in Rural Areas: Evidence from Southern Jordan

**DOI:** 10.3390/antibiotics15010004

**Published:** 2025-12-19

**Authors:** Anas Khaleel, Anwar Ali Al-Shamaileh, Mohammad Ameen Al-Aghbar, Wael Abu Dayyih, Suhaib Muflih, Haneen Aljamal, Ahmed S. A. Ali Agha, Mohammad Hailat, Ahmad Al Athamneh

**Affiliations:** 1 Faculty of Pharmacy and Medical Sciences, University of Petra, Amman 11196, Jordan; ahmad.alathamneh@uop.edu.jo; 2 Department of Pharmaceutical Chemistry, Faculty of Pharmacy, Mutah University, Al-Karak 61710, Jordan; tarawnehanwar82@gmail.com; 3 Department of Medical Laboratory Sciences, Faculty of Allied Medical Sciences, Al-Ahliyya Amman University, Amman 19111, Jordan; 4 Department of Pharmacy Practice, College of Pharmacy, Imam Abdulrahman Bin Faisal University, Dammam 31441, Saudi Arabia; 5 School of Pharmacy, Department of Clinical Pharmacy, The Jordan University of Science and Technology, Irbid 22110, Jordan; 6 School of Pharmacy, Department of Pharmaceutical Sciences, The University of Jordan, Amman 11942, Jordan; 7 Faculty of Pharmacy, Al-Zaytoonah University of Jordan, Amman 11733, Jordan; m.hailat@zuj.edu.jo

**Keywords:** non-prescription antibiotics, antimicrobial resistance (AMR), community pharmacists, knowledge-attitude-practice (KAP), antibiotic dispensing, antimicrobial stewardship (AMS)

## Abstract

**Background/Objectives:** Antimicrobial resistance (AMR) is a major global health issue. Since community pharmacists are frontline health officials regarding the provision and management of antibiotics, it is of great importance to study the knowledge, attitudes, and practices (KAPs) of pharmacists with respect to antimicrobial stewardship (AMS) to formulate specific interventions. In Jordan, where dispensing antibiotics without a prescription is a common situation, this study was designed to assess the KAPs of community pharmacists in southern Jordan on AMR and AMS, the gap in the knowledge base and the practice, and the effect of other variables on antimicrobial education and responsible dispensing. **Results:** Participant pharmacists (n = 383) confirmed a moderate or lack of knowledge in antibiotic choice, resistance mechanisms, and the basis of stewardship. Despite positive attitudes for AMS, important practice gaps occurred: 38.6% infrequently dispensed antibiotics without prescriptions, 67.4% mistakenly believed that antibiotics may cure viral infections (flu/common cold), and only 33.4% firmly rejected non-prescription antibiotic requests. Knowledge scores were significantly higher among bachelor’s degree pharmacists, public university pharmacists, and urban-working pharmacists. Practice scores were better among master’s degree holders and urban practitioners. **Conclusions:** This work indicates that the AMS knowledge and practices of pharmacists in the southern Jordanian community are lacking in a severe way, with almost 4 out of 10 having poor behaviors including dispensing non-prescription antibiotics.

## 1. Introduction

Antimicrobial resistance (AMR) is one of the most urgent international health issues of the 21st century, putting decades of medical advances in the fight against infectious diseases. Inappropriate and overuse of antibiotics, the lack of public education, and the absence of antimicrobial stewardship (AMS) initiatives have increased the frequency and rate of resistant pathogen emergence and spread all over the globe. In 2019, the global death toll directly related to bacterial AMR was estimated at 1.27 million, with nearly 5 million deaths being related to resistant infections [1]. Many developing countries encounter disparate burdens due to inadequate regulatory frameworks, insufficient infection control infrastructure, and extensive dispensing of antibiotics without prescriptions [2].

The financial effect of AMR is also overwhelming. In the US, it is estimated that approximately 35,000 people die and 2.8 million people are infected each year by antibiotic-resistant infections, and there is a direct healthcare expenditure of greater than USD 4.6 billion [3]. By 2030, AMR may decrease the gross domestic product by 1.138 percent and cost over USD 1 trillion a year if the current trends remain unchecked worldwide [4]. These practices highlight the need to have effective antimicrobial stewardship interventions in every healthcare setting.

AMR is a growing public health concern caused by several interrelated factors. In the Jordanian healthcare system, community pharmacies are privately owned, frontline healthcare outlets that provide direct, walk-in access to medications. With a higher role in rural communities, community pharmacists contribute to battling AMR, as the proper use of antibiotics allows a high rate of self-treatment, and most patients end up abusing antibiotics without consulting professionals [5]. The situation of free access to antibiotics in most low- and middle-income countries (LMICs) revolves around the professional gatekeeping process, resulting in direct purchasing without prescriptions. Inadequate education of healthcare professionals in AMS programs exacerbates the problem [2]. Additionally, the development of new antibiotics has drastically reduced since the 1980s because of the high expenses involved in research and development, and the low profits generated by the investments. This situation was accompanied by the dramatic increase in multi-drug-resistant bacteria, fungi, and parasites. Bacteria develop advanced modes of resistance, such as enzymatic breakdown of drugs, drug target modification, induction of efflux pumps, and reduced membrane permeability [6]. These responses are the results of selection pressure from exposure to antibiotics, and they demonstrate the necessity of strong AMS measures to reduce the rate at which resistant bacteria develop and spread.

Initiatives by the WHO in AMR surveillance led to a global action plan that was announced in 2015, and emphasized the need for international cooperation, robust surveillance systems, and multisectoral interventions [1,7]. However, this is still not implemented in most resource-limited countries. Community pharmacists, therefore, have a special role as easily accessible providers of healthcare and may be the initial contact for patients exhibiting minor conditions [8]. The gatekeeping role that pharmacists play is especially important in most LMICs due to the prevalence of easy access to non-prescription antibiotics [9].

Little research has been done to determine the AMS competencies and practices of community pharmacists in developing nations. Research conducted in different regions indicates that there are significant knowledge gaps, a lack of consistency in the attitudes towards stewardship principles, and inefficient dispensing practices [10,11]. High-income nations have demonstrated that pharmacist-led interventions can significantly enhance pharmacist-to-patient relationships and reduce resistance rates [12,13]. Nonetheless, to apply these models in LMIC settings, it is necessary to learn about the local barriers, facilitators, and contextual factors that may shape the behavior of pharmacists.

AMR challenges are high in Jordan, which is one of the middle-income countries in the Eastern Mediterranean Region. Jordan is also among the highest rates in the region for antibiotic usage, estimated at about 18.8 defined daily doses per a thousand inhabitants per day [14]. Resistant infections acquired in the community are increasingly becoming a risk to the health of the people. Although the law mandates that prescriptions are taken, it is inconsistently enforced, and antibiotics are usually bought over the counter in local pharmacies [15].

The pharmacy education environment in Jordan comprises 18 accredited institutions that provide Bachelor of Pharmacy (PharmC, 5 years) and Doctor of Pharmacy (PharmD, 6 years) degrees. Nevertheless, the content of AMS differs among the curricula, and there are no standard competency requirements [16]. Professional development opportunities on antimicrobial stewardship are also still rare, especially beyond large urban centers. Southern Jordan faces distinct healthcare challenges that complicate antimicrobial stewardship efforts. These structural barriers create an environment where optimal antimicrobial use is systematically more difficult to achieve compared to northern urban centers, making this region particularly important for targeted AMS interventions.

The KAP framework is a systematic way of evaluating the capabilities of medical professionals and determining areas of intervention [17]. Knowledge will encompass a comprehensive understanding of AMR mechanisms and stewardship principles. Attitudes display beliefs, perceptions, and values about antimicrobial use and resistance. Practice reflects real actions of dispensing decisions, counseling patients, and compliance. Although the KAP model presupposes knowledge as an influencing factor on attitudes, the linear relationship is frequently more complicated. The knowledge-to-practice scheme may be disrupted by external factors such as regulatory enforcement, workplace culture, patient expectations, economic pressures, and aggregate barriers [18].

This report has contributed significantly to current research by revealing the complete KAP assessment of community pharmacists in southern rural areas of Jordan, detecting detailed knowledge and practice gaps that require urgent consideration, and enlightening barriers and facilitators of optimum stewardship practices.

## 2. Results

### 2.1. Participant Demographics

The study included 383 community pharmacists with a mean age of 29.9 years (SD = 7.81, range 22–58). The majority were female (69.2%, n = 265), younger than 30 years (65.8%, n = 252), and had a mean pharmacy experience of 6.0 years (SD = 5.23, range 1–35). Most participants held bachelor’s degrees (68.9%, n = 264), graduated from public universities (73.9%, n = 283), were recent graduates (66.1% from 2015–2024 cohort), worked as staff pharmacists (54.8%, n = 210), and practiced in urban areas (79.4%, n = 304). Pharmacy owners comprised 28.7% (n = 110) and managers 16.4% (n = 63) of the sample. Complete demographic characteristics are presented in Table 1.

### 2.2. Instrument Reliability

Cronbach’s alpha values confirmed acceptable internal consistency: Knowledge α = 0.787 (23 items), Attitude α = 0.780 (18 items), and Practice α = 0.692 (20 items). Test–retest reliability coefficients demonstrated adequate temporal stability: Knowledge r = 0.73, Attitude r = 0.70, and Practice r = 0.92 (all *p* < 0.001). Intraclass correlation coefficients for individual items ranged from 0.58 to 0.89, confirming moderate-to-good reliability for most questions.

### 2.3. Knowledge of Antimicrobial Use and Resistance

Knowledge scores ranged from 42.5 to 64.2 points out of a maximum 69, with a mean of 52.5 (SD = 6.84, representing 76.1% accuracy) and median of 53.0 (IQR = 48.0–57.0). Using established cut-offs, 76.2% (n = 292) demonstrated high knowledge (47–69 points) and 23.8% (n = 91) moderate knowledge (24–46 points). No participants scored in the low knowledge category (<24 points).

Despite reasonable overall knowledge, critical deficiencies emerged. Table 2 presents detailed response distributions for selected knowledge items. Key gaps included widespread uncertainty about viral infections: 67.4% (n = 258) were uncertain whether antibiotics effectively treat common colds, coughs, and the flu, while only 9.1% (n = 35) correctly identified this as false and 23.5% (n = 90) incorrectly believed that antibiotics help with treating viral infections. Similarly, 75.7% (n = 290) expressed uncertainty about antibiotic effectiveness against viruses, with only 8.6% (n = 33) correctly answering “no.”

**Figure 1:**
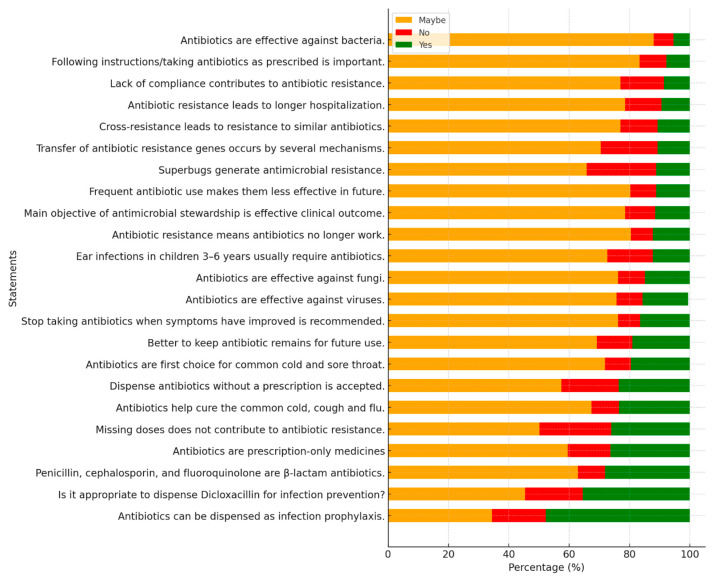
Stacked bar chart illustrating pharmacists’ self-perceived knowledge on various aspects of antimicrobial use and resistance. The responses are categorized as ‘Maybe’ (orange), ‘No’ (red), and ‘Yes’ (green) for each statement. The data reflects the level of understanding and misconceptions regarding antibiotic use, effectiveness, and resistance mechanisms.

Knowledge about resistance mechanisms was also deficient. While 80.4% (n = 308) correctly understood that antibiotic resistance means antibiotics no longer work effectively, and 80.2% (n = 307) recognized that frequent unnecessary use makes antibiotics less effective in the future, half of the participants (50.1%, n = 192) incorrectly believed that missing one or two antibiotic doses does not contribute to resistance development, with only 23.8% (n = 91) correctly disagreeing and 26.1% (n = 100) answering that they were uncertain. Furthermore, only 28.2% could correctly identify beta-lactams as one of the major antibiotic classes, indicating substantial gaps in foundational pharmacology knowledge (Table 2, and Figure 1).

**Figure 2:**
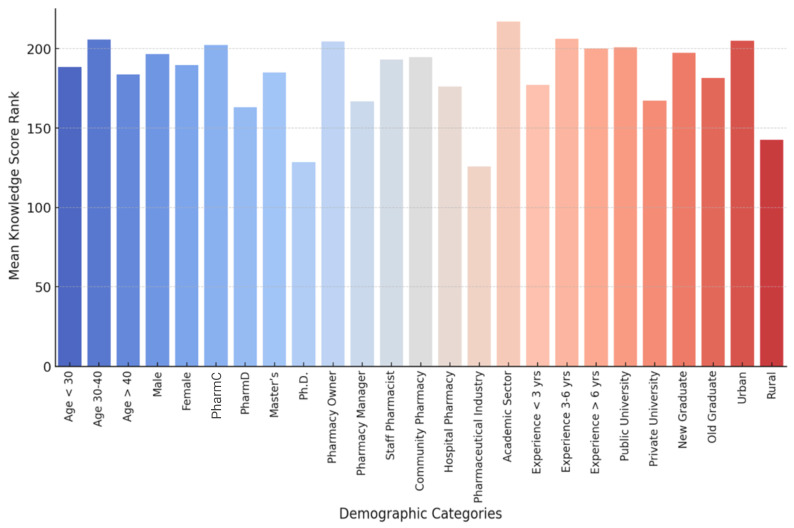
Comparison of knowledge scores by education level and work location. The box plot illustrates the distribution of knowledge scores (mean rank) across different demographic categories, including education level, work experience, and work location.

No significant results were obtained in the statistical analyses of knowledge scores with respect to age (*p* = 0.387), gender (*p* = 0.587), and job position (*p* = 0.094). Nevertheless, the educational level showed a significant association (*p* = 0.019), as the highest score was observed in the case of the academic level of the PharmC degree holders (median = 54.0), PharmD (median = 51.0), Master’s degree (median = 53.0), and Ph.D. (median = 52.5). There was also a strong correlation with university type (*p* = 0.009, r = 0.15), with higher scores among the public university graduates (median = 54.0) than the graduates from private universities (median = 51.0). Most notably, practice location demonstrated a strong association (*p* < 0.001, r = 0.28), with urban pharmacists scoring substantially higher (median = 54.0, IQR = 50.0–58.0) than rural pharmacists (median = 48.0, IQR = 45.0–52.0) (Figure 2).

### 2.4. Attitudes Toward Antimicrobial Use and Stewardship

The median attitude score was 4.0 (IQR = 3.5–4.5) on the 5-point Likert scale, indicating generally positive attitudes toward antimicrobial stewardship. Pharmacists demonstrated contradictory attitudes regarding non-prescription dispensing. While one-third (33.4%, n = 128) strongly disagreed that dispensing antibiotics without prescriptions is acceptable, nearly an equal proportion (31.3%, n = 120 for combined responses of “strongly agree” and “agree”) found this practice acceptable. Furthermore, 44.9% (n = 172 for combined responses of “strongly agree” and “agree”) believed that they possessed sufficient knowledge to assess patients and dispense antibiotics without prescriptions after self-evaluation.

Regarding AMR awareness, less than half (42.3%, n = 162) agreed that AMR represents an important public health problem, though a slim majority (50.4%, n = 193 for combined responses of “strongly agree” and “agree”) acknowledged pharmacists’ professional responsibility for reducing resistance. Economic considerations emerged as a substantial barrier: 53.0% (n = 203 for combined responses of “strongly agree” and “agree”) acknowledged that the prohibition of non-prescription antibiotic sales would negatively impact pharmacy revenues and profits. In the One Health domain, only one-third (33.7%) recognized the contribution of veterinary antibiotic use to human antimicrobial resistance.

**Figure 3:**
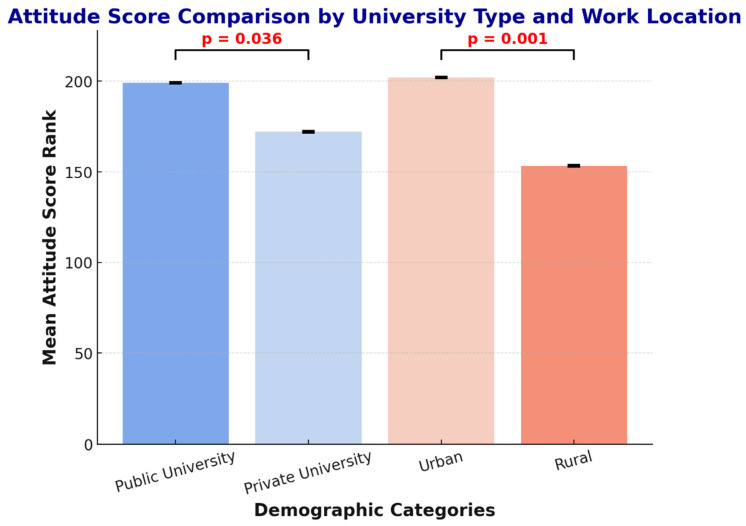
Comparison of attitude scores by university type and work location. The figure illustrates the mean attitude score ranks across demographic categories. Public university graduates exhibited significantly higher attitude scores (*p* = 0.036) compared to private university graduates, while urban pharmacists demonstrated significantly more positive attitudes (*p* < 0.001) than their rural counterparts. Significance brackets indicate statistically significant differences.

The statistical analysis revealed no significant associations between attitude scores and age (*p* = 0.100), gender (*p* = 0.206), or education level (*p* = 0.201). However, significant associations emerged for university type (*p* = 0.036, r = 0.12), with public university graduates demonstrating more positive attitudes (median = 4.1) compared to private university graduates (median = 3.8). Practice location also showed a strong association (*p* < 0.001, r = 0.25), with urban pharmacists exhibiting significantly more favorable attitudes (median = 4.2, IQR = 3.7–4.6) than rural pharmacists (median = 3.6, IQR = 3.2–4.0) (Figure 3).

### 2.5. Practices in Antimicrobial Use and Stewardship

The median practice score was 4.0 (IQR = 3.6–4.3) on the 5-point frequency scale. Table 3 presents the response distributions for selected practice items.

There are positive aspects of the patient-centered care that the pharmacists exhibited. Most of them reported constantly or often evaluating the signs and symptoms of the patients before giving them antibiotics, requesting information about drug interactions, adverse reactions, and allergies, and instructing patients on the proper use and resistance of antimicrobials. Pediatric dose verification was almost universal, as 81.5% (n = 312) of them stated that they always or frequently calculated and verified appropriate dosages in children.

Nevertheless, critical practice gaps developed. The most worrying was general dis-prescription dispensing: 38.6% (n = 148) reported having sometimes dispensed antibiotics without prescriptions when they encountered similar clinical presentations, and this percentage rose to 60.6 when the participants were specifically questioned about the past month; many of them agreed that they had dispensed antibiotics without prescription at least sometimes (31.9% “sometimes”, 9.4% often, 19.3% always).

Guideline observance was below optimal: only 52.2% (n = 200 for combined responses of “often” and “always”) regularly checked antimicrobial prescriptions according to therapeutic guidelines before dispensing, while 37.3% (n = 143) did so only “sometimes.” Interprofessional collaboration remained limited, with less than half (43.3%, n = 166 for combined responses of “often” and “always”) reporting frequent cooperation with physicians, nurses, or other healthcare professionals for infection control and antimicrobial stewardship initiatives.

**Figure 4:**
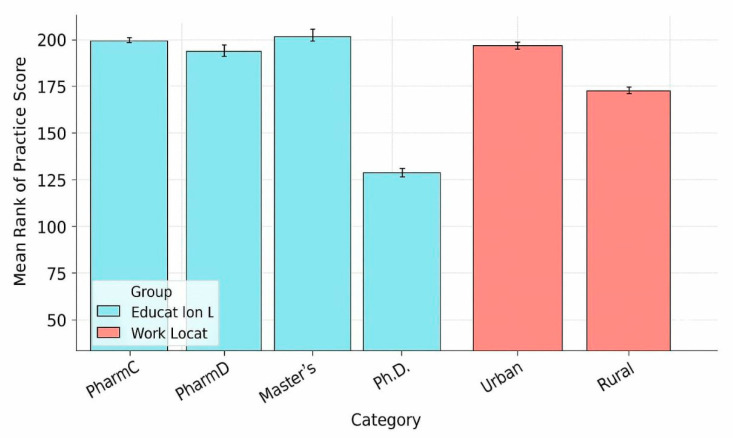
Distribution of mean rank practice scores by education level and work location. Blue bars represent education level (PharmC, PharmD, Master’s, Ph.D.), showing Master’s degree holders with the highest scores. Red bars represent work location, with urban pharmacists scoring higher than rural counterparts. Both factors showed significant associations with practice scores (education: *p* = 0.040; location: *p* = 0.048).

Statistical analyses revealed no significant associations between practice scores and age (*p* = 0.412), gender (*p* = 0.673), or job position (*p* = 0.158). However, two factors demonstrated significant associations: education level (*p* = 0.040), where pharmacists holding Master’s degrees exhibited significantly better practices (median = 4.3) compared to Bachelor’s PharmC (median = 4.0), PharmD (median = 3.9), or Ph.D. holders (median = 4.1. Figure 4). The other factor is practice location (*p* = 0.048, r = 0.11), with urban pharmacists demonstrating superior practices (median = 4.1, IQR = 3.7–4.4) compared to their rural counterparts (median = 3.8, IQR = 3.4–4.1).

### 2.6. Correlations Between Knowledge, Attitude, and Practice

Spearman’s correlations between KAP dimensions were all positive but non-significant: Knowledge–Attitude rs = 0.027 (*p* = 0.629); Attitude–Practice rs = 0.072 (*p* = 0.182); and Knowledge–Practice rs = 0.081 (*p* = 0.132). These non-substantial associations show that pharmacists’ knowledge levels did not affect their attitudes, and neither knowledge nor attitudes strongly predict the described practices, signifying that external aspects override individual capabilities in defining professional behavior.

### 2.7. Multiple Regression Analysis

**Knowledge Predictors:** The multiple linear regression model (F(6, 376) = 5.84, *p* < 0.001, R^2^ = 0.085) identified practice location (urban) (B = 3.42, β = 0.24, *p* < 0.001) and university type (public) (B = 1.87, β = 0.13, *p* = 0.010) as significant independent predictors, explaining 8.5% of the variance in knowledge scores.

**Attitude Predictors:** The model (F(2, 380) = 12.43, *p* < 0.001, R^2^ = 0.061) identified practice location (B = 0.48, β = 0.21, *p* < 0.001) and university type (B = 0.31, β = 0.12, *p* = 0.018) as significant predictors, explaining 6.1% of the variance.

**Practice Predictors:** The model (F(4, 378) = 2.78, *p* = 0.027, R^2^ = 0.028) explained only 2.8% of variance, with Master’s degree (B = 0.34, β = 0.09, *p* = 0.044) and urban location (B = 0.27, β = 0.11, *p* = 0.030) as significant predictors. The low R^2^ indicates that most practice variability stems from unmeasured external factors.

## 3. Discussion

This assessment of 383 community pharmacists in southern Jordan found that there was a contradiction between the moderate accuracy of the knowledge (76%) and the generally positive attitudes towards antimicrobial stewardship, but critical knowledge gaps and practice behaviors were identified. Almost 40 percent of the participants reported dispensing antibiotics without a prescription, although it is prohibited by law. Most importantly, there were no significant correlations between knowledge, attitudes, and practices, which revealed that external factors, rather than individual competencies, are the significant determinants of professional behavior.

The mean knowledge score of 76% is similar to that in nearby Middle Eastern nations, including Saudi Arabia (72%), Lebanon (78%), and Pakistan (71%), but inferior to high-income nations like Australia (86%) and the United Kingdom (84%). This pattern suggests that educational quality, access to continuing education, and practice environments in LMICs systematically affect knowledge acquisition and retention [10,19].

Problematic myths were formed that have immediate practical implications. Two-thirds of the participants did not know whether antibiotics are used to treat viral infections; a fundamental deficiency that probably leads to the inappropriate dispensing of cold and flu medications. Such misunderstandings have been reported in all developing nations, and the issue is a worldwide problem with education that needs changes in the curricula [5,20]. The understanding of AMR mechanisms was also very poor, with less than 15 percent of participants understanding the resistance development pathways concerning dose completion. The lack of this is also indicated by results in India and Kenya [20], where more emphasis on complex microbiological concepts is not received in pharmacy education.

Surprisingly, PharmC degree holders performed better than PharmD graduates in knowledge tests. This unexpected finding can be explained by several reasons. First, the knowledge question focused on factual recollection rather than clinical reasoning, an area where PharmD preparation is particularly strong. Second, the more recent PharmD programs in Jordan might be lagging behind the established content integration in the longstanding PharmC programs. Third, not all PharmD graduates follow a community practice route, and instead may follow a hospital route, which may influence their interaction with community-specific issues relating to AMS. The higher knowledge of public university graduates may be explained by the fact that older programs, with more experienced faculty and increased resources, are found in the flagship institutions of Jordan.

Our results align with and support the recent national data from Al-Taani et al. (2022) [21]. Both studies showed that community pharmacists have a similar level of knowledge and a similar level of non-prescription dispensing. However, some essential distinctions become apparent in terms of regional patterns that were categorized: first, the urban–rural knowledge gap in our southern sample (urban: 54.0 median score vs. rural: 48.0, *p* < 0.001) was more pronounced than national patterns. Second, information source accessibility is poor, as while Al-Taani et al. [21] found that 43.1% of pharmacists nationally accessed continuing education programs, our informal follow-up queries suggest that <20% of rural southern pharmacists have regular access to such programs. Third, practice location emerged as the strongest predictor in our southern sample (explaining 8.5% of knowledge variance), suggesting that geographic isolation compounds educational barriers. Moreover, the gap between poverty levels (rural 25%, national scale 14%) and population size (rural 52%, national scale 15%), as well as variations in the healthcare infrastructure, were not addressed. Therefore, such structural inequalities may tend to create systemic disparities that obstruct ideal antimicrobial stewardship in rural areas.

The non-prescription medication dispensing rate of 38.6 percent in our case study in the southern region of Jordan places this region in the middle of dispensing practices in the Middle East. The given rate is higher than the statistics in Pakistan (71%) [10], Egypt (71%) [22], Syria (67%) [9], Lebanon (52%) [23], and Saudia Arabia (45–48%) [24], but it is, nonetheless, significantly higher than that of the United Arab Emirates (15%) [9], where there is electronic prescription monitoring and strong enforcement in place. This is a comparative analysis that points out that dispensing behavior is predominantly controlled by regulatory infrastructure as opposed to a single cultural determinant or the competence of the pharmacist (Table 4 summarizes these studies).

The accuracy of the knowledge of the physicians, which was at 76.1%, is in tandem with the accuracy in other jurisdictions of the Middle East, which includes Lebanon, Saudi Arabia, and Pakistan at 78, 72, and 71, respectively. It is, however, poor in comparison with other high-income countries like Australia (86%) [25]. This result suggests a systematic difference in the quality of pharmacy education, the availability of continuing professional growth, and practice environment support, but not a difference in individual motivations or competence.

The considerable knowledge gap in urban–rural (*p* = 0.001, moderate effect size = 0.28) is a significant impartiality concern. Compounding has been disadvantageous to rural pharmacists: they have limited access to continuing education, are professionally isolated, and they have fewer diverse clinical cases, fewer resources, and less interaction with academic centers. This inequality is typical of the global trends reported in many other countries and requires specific interventions such as technology-based learning platforms, subsidies to fund the provision of rural pharmacists with training programs, urban–rural mentorships that match the experienced practitioners with rural practitioners, and mobile learning teams that provide on-site training [26].

For practicing pharmacists, continuing education should employ evidence-based approaches including audit-and-feedback on individual dispensing patterns compared to peers and guidelines, academic detailing through individualized visits by trained educators, and communities of practice fostering peer learning networks [27]. Pharmacists’ generally positive attitudes toward stewardship (median 4/5) align with regional studies from Lebanon, Pakistan, and Saudi Arabia [10,19,23], suggesting widespread recognition of the importance of AMR. However, these positive attitudes failed to translate to behavior, as evidenced by non-significant attitude–practice correlations (rs = 0.072, *p* = 0.182). This attitude–behavior gap is well-documented in health psychology literature and indicates that intentions and beliefs are necessary but insufficient for behavior change [28].

A remarkable contradiction occurred: while 40.7% assumed that pharmacists should discontinue non-prescription dispensing, 31.3% found it acceptable, and 38.6% engaged in the practice. This intellectual disagreement, which requires holding contradictory convictions, likely stems from justifications such as “patients need access,” “I can evaluate appropriately given my training,” or “economic survival requires meeting customer demands”. In this regard, ethical disengagement allows professionals to reconcile their practices, negating these adopted values [29].

The most interesting observation in this study is that knowledge, attitudes, and practices did not have any significant correlations (all *p* > 0.10). This is in very sharp contrast to the assumption of the traditional KAP model that attitudes are determined by knowledge, which governs practices. Although the framework has a handy structure to use in assessments, it is linear in nature, and complex behavioral determinants are ignored by this structure. The observation is consistent with accumulating evidence that knowledge has little behavioral impact, which is recorded in systematic reviews in healthcare environments [30].

Various outside influences are also likely to dominate single competencies. Laxity in the enforcement of regulations eliminates the accountability of non-compliant dispensing. Behavioral economics illustrates the fact that tangible reinforcements here and now (sales made) have a more decisive impact than delayed abstract reinforcements (decreased community resistance rate) [31].

The low R^2^ of the regression analysis with practice scores (0.028) validates the fact that the measured demographic characteristics produce very little variance. Unmeasured systemic variables, such as workplace policy, patient demand patterns, economic incentives, and the consistency of regulatory enforcement, are the main sources of practice variability. The systematic reviews prove that multifaceted interventions that target structural obstacles, environmental facilitators, and systems of accountability are more effective than education-only interventions [32,33].

Formal integration of community pharmacists into antimicrobial stewardship programs with specific roles, channels of communication through which they can consult prescribers, and access to diagnostic assistance where possible would make practices more potent at the health-system level. Policy-wise, there should be enhanced regulatory enforcement and effective penalties based on the severity of the violation, the creation and publication of national AMS guidelines adapted to the community pharmacy environment, economic incentives to reward the proper prescribing patterns and compliance with the guidelines, and the use of electronic prescription monitors that would allow for tracking the patterns of antibiotic dispensing in real-time [34,35].

Discrepancies in resource distribution, uneven funding directives for underserved areas, internet connectivity financing for training development, and free access to online clinical databases and guideline resources represent necessary investments. Regionalized AMS programs, determining regional antimicrobial stewardship centers with local campaigns, accessible telephone consultation services, and regular case review discussions, can provide ongoing reinforcement [36].

### Study Limitations

Several limitations should be mentioned when the findings are interpreted. The cross-sectional design does not permit causal conclusions; we can only conclude associations but cannot determine whether knowledge deficits are the cause of poor practices or the result of common underlying factors. The underreporting of non-prescription dispensing may have occurred due to the bias of self-reporting, which allows for social desirability; yet, the prevalence of inappropriate practices was quite significant, indicating a reasonable level of candor. The knowledge test was based on self-perceived knowledge as opposed to clinical competency testing based on objectively administered standardized patients or practice audits.

Many possibly significant variables were not measured, such as pharmacy ownership structure (independent or chain), prescription volume and complexity, staffing ratios, locality to hospitals or clinics, and local AMR surveillance data. Qualitative research designs would yield a deeper insight into situational factors, decision-making processes, and the experience of living with competing forces between professional ethics and survival in the economy.

## 4. Materials and Methods

### 4.1. Study Design and Setting

The present study was a cross-sectional, descriptive study carried out between March and August 2024 in the southern governorates of Jordan, namely Ma’an, Tafilah, Aqaba, Karak, and a section of Balqa, among community pharmacists in the specified areas. Prior to the main survey, the questionnaire was piloted with 15 community pharmacists to assess clarity, relevance, and feasibility, and minor adjustments were made accordingly.

### 4.2. Ethical Approval

The study was approved by the Institutional Review Board of Mutah University (MUTAH-2023/A1, approved 15 January 2024) and was executed following the principles of the Declaration of Helsinki. Electronic informed consent was given by all the respondents prior to filling in the questionnaire.

### 4.3. Study Population and Sample Size

The target participants were the licensed community pharmacists who were practicing in the south of Jordan. The inclusion criteria were as follows: (1) Pharmacy degree (PharmC, PharmD, Master, or Ph.D.); (2) currently working in a community pharmacy environment; (3) currently working in one of the specified southern governorates; and (4) informed consent. Exclusion criteria were non-practicing pharmacists, hospital or clinical pharmacists and non-consenting ones.

The sample size was determined using the Raosoft online calculator with 95 percent confidence gap, 5 percent margin of error, and population of 1500 community pharmacists. Finally, 383 complete answers were received, which is sufficient to have statistical power to conduct all the intended analyses.

### 4.4. Sampling Strategy

The sampling method used was two-stage sampling. To begin with, the lists of registered community pharmacies were received via official requests to the Jordanian Pharmacists Syndicate and the Ministry of Health. Second, the governorate used a stratified random sampling method to represent proportionality. Also, snowball convenience sampling was used as an adjunct to the random sample by use of professional social media groups and direct contacts. As much as this creates a possibility of selection bias, the demographic distribution of the final sample was similar to that of the national data, implying that the sample was well represented.

### 4.5. Questionnaire Development

The research instrument was a structured, self-administered questionnaire, which was formulated by a systematic literature review on AMR and AMS measurement instruments in published literature [10,11]. This was based on the national level of the challenge assessment performed by Al-Taani et al. (2022) [21]. We tailored our questionnaire to respond to the unique set of challenges experienced in the rural communities of southern Jordan, which may be facing more limited access to continuing education and clinical resources than northern urban areas. The questionnaire was localized in the context of the pharmacy community in Jordan and had four sections:

1: Demographics (9 items): age, gender, education level, job position, working field, experience in pharmacy, type of university (public/private), graduation year, and practice location (urban/rural).
2: Knowledge Assessment (23 items): assessing the knowledge related to antimicrobial resistance by using binary and multiple-answer questions about basic antibiotic pharmacology, mechanisms, transmission of resistance, indications of appropriate prescribing, and principles of stewardship. The items were rated as correct (3 points), incorrect (0 points), or uncertain (1 point). The total knowledge scores were between 0 and 69 points.
3: Attitude Assessment (18 items): the attitude to AMR as a public health problem, the professional roles of pharmacists, the suitability of dispensing non-prescription drugs, economic benefits, and the desire to engage in stewardship activities, assessed with 5-point Likert scales (1 = Strongly Disagree to 5 = Strongly Agree).
4: Practice Assessment (20 items): assessment of dispensing practices via the use of 5-point frequency scales (1 = Never to 5 = Always) of patient assessment before dispensing, prescription verification, patient counseling on antimicrobial use and resistance, collaboration with healthcare professionals, guideline adherence, and real non-prescription dispensing frequency. The questionnaire was translated into Arabic by two independent bilingual pharmacists with back-translation to verify semantic equivalence. Face validity was established through expert panel review by 12 clinical pharmacists and pharmacy practice faculty members. A pilot study with 15 community pharmacists was conducted to refine item clarity based on participant feedback.

### 4.6. Reliability and Validity Assessment

**Internal Consistency:** Cronbach’s alpha coefficients demonstrated acceptable reliability: Knowledge α = 0.787, Attitude α = 0.780, and Practice α = 0.692. These values surpass the generally accepted threshold of 0.70 for newly established questionnaires.

**Test-Retest Reliability**: The questionnaire was administered twice to thirty pharmacists with a one-week delay between the two sessions, and the Pearson correlations are as follows: Knowledge r = 0.73, Attitude r = 0.70, and Practice r = 0.92, which all indicate good temporal stability.

**Content Validity:** The questionnaire was checked by five independent experts with the help of the content validity index (CVI) assessment. The questionnaire had an item-level CVI (I-CVI) of above 0.78 and a scale-level CVI of 0.89, which has shown excellent content validity [37].

### 4.7. Data Collection

The survey was conducted online through Google Forms between March and August 2024. Distribution channels were direct emails to randomly chosen pharmacies, professional social media groups (Jordanian Pharmacists Association Facebook groups), WhatsApp networks, and distribution of QR codes in person when visiting the pharmacy. The survey introduction page contained information about the study in the form of detailed information, and assured confidentiality and anonymity. Electronic informed consent was obtained before the participants were allowed to view the questionnaire. No personal identifying information was collected, and three follow-up reminders were sent at two-week intervals to maximize response rate. Of 428 initiated responses, 383 (89.5%) were sufficiently complete (>90% of items answered) for inclusion in final analyses.

### 4.8. Statistical Analysis

Data was analyzed using IBM SPSS Statistics Version 28.0 (IBM Corp., Armonk, NY, USA). Descriptive statistics summarized demographic characteristics and KAP variables using frequencies, percentages, means, standard deviations, medians, and interquartile ranges as appropriate. One-sample Kolmogorov–Smirnov tests assessed distribution normality for continuous variables. Due to non-normal distributions for attitude and practice scores (both *p* < 0.001), non-parametric tests were employed throughout: Mann–Whitney U tests compared two independent groups, Kruskal–Wallis H tests compared three or more groups with post-hoc pairwise comparisons when significant, and Spearman’s rank correlation coefficients (rs) assessed relationships between KAP dimensions. Multiple linear regression models identified independent predictors of KAP scores after checking assumptions including linearity, homoscedasticity, independence of residuals, and absence of multicollinearity (variance inflation factor < 5). Statistical significance was set at *p* < 0.05 (two-tailed), with effect sizes reported alongside *p*-values to provide complete interpretation [38,39].

## 5. Conclusions

This systematic report identified severe gaps in the knowledge of antimicrobial stewardship and practices in rural places in southern Jordan, and a high rate of non-prescription dispensing of antibiotics despite, in general, favorable views of the concept of antimicrobial stewardship.

Taking into consideration all these overall findings, we suggest combined recommendations to various stakeholder groups. During the education of pharmacists, it is recommended that the curriculum emphasize AMS skills and knowledge with greater foundations and underpinnings in microbiology, and that One Health concepts are taught that link human, animal, and environmental health. Engagement in antimicrobial stewardship programs should focus on pedagogical strategies, which incorporate active learning techniques other than standard lectures, such as simulation, case discussions, and experiential rotations. Before graduation, AMS competencies must be checked through competency-based testing using the administration of objective structured clinical examinations (OSCEs) and performance testing based on the workplace.

## Figures and Tables

**Table 1:** Demographic characteristics of study participants (N = 383).

Characteristic	n (%)	Mean ± SD
**Age (years)**		29.9 ± 7.81
<30 years	252 (65.8)	
30–40 years	91 (23.8)	
>40 years	40 (10.4)	
**Gender**		
Female	265 (69.2)	
Male	118 (30.8)	
**Pharmacy Experience (years)**		6.01 ± 5.23
<3 years	157 (41.0)	
3–6 years	85 (22.2)	
>6 years	141 (36.8)	
**Highest Qualification**		
Bachelor of Pharmacy (PharmC)	264 (68.9)	
Doctor of Pharmacy (PharmD)	60 (15.7)	
Master’s Degree	49 (12.8)	
Ph.D. Degree	10 (2.6)	
**Job Position**		
Pharmacy Owner	110 (28.7)	
Pharmacy Manager	63 (16.4)	
Staff Pharmacist	210 (54.8)	
**University Type**		
Public University	283 (73.9)	
Private University	100 (26.1)	
**Graduation Cohort**		
Recent (2015–2024)	253 (66.1)	
Older (Before 2015)	130 (33.9)	
**Practice Location**		
Urban	304 (79.4)	
Rural	79 (20.6)	

**Table 2:** Pharmacists’ knowledge of antimicrobial use and resistance (N = 383).

Statement	Strongly Agree n (%)	Agree n (%)	Neutral n (%)	Disagree n (%)	Strongly Disagree n (%)	Median
It is okay to dispense antibiotics without prescription	22 (5.7)	98 (25.6)	84 (21.9)	51 (13.3)	128 (33.4)	4
Pharmacists are knowledgeable enough to dispense without Rx after assessment	69 (18.0)	103 (26.9)	92 (24.0)	98 (25.6)	21 (5.5)	3
AMR is an important public health problem	22 (5.7)	162 (42.3)	43 (11.2)	18 (4.7)	138 (36.0)	4
Pharmacists are responsible for reducing resistance	16 (4.2)	177 (46.2)	55 (14.4)	18 (4.7)	117 (30.5)	4
Prohibition will decrease sales and profits	63 (16.4)	140 (36.6)	93 (24.3)	57 (14.9)	30 (7.8)	3

**Table 3:** Pharmacists’ practices regarding antimicrobial use—selected items (n = 383).

Practice Statement	Never n (%)	Rarely n (%)	Sometimes n (%)	Often n (%)	Always n (%)	Median
Ask about signs/symptoms before dispensing antibiotics	17 (4.4)	22 (5.7)	61 (15.9)	28 (7.3)	255 (66.6)	4
Screen antibiotics according to therapeutic guidelines.	8 (2.1)	32 (8.4)	143 (37.3)	28 (7.3)	172 (44.9)	4
Instruct patients on antimicrobial use and resistance.	14 (3.7)	33 (8.6)	75 (19.6)	34 (8.9)	227 (59.3)	4
If dealing with a similar case, may dispense without a prescription.	82 (21.4)	66 (17.2)	148 (38.6)	35 (9.1)	52 (13.6)	3
In last month, dispensed antibiotics without Rx.	72 (18.8)	79 (20.6)	122 (31.9)	36 (9.4)	74 (19.3)	3
When dispensing for children, calculate dose.	8 (2.1)	13 (3.4)	50 (13.1)	54 (14.1)	258 (67.4)	4
Collaborate with healthcare professionals for AMS.	13 (3.4)	66 (17.2)	138 (36.0)	43 (11.2)	123 (32.1)	3

**Table 4:** Evaluation of community pharmacist antimicrobial resistance KAP across Middle East nations and select high-income countries.

Country	Study	Sample Size	Knowledge Level	Non-Prescription Dispensing Rate	Key Factors
Middle East					
Jordan (South)	Khaleel et al. 2025 (current study)	383	76% accuracy	38.6%	Rural isolation, economic pressure
Jordan (National)	Al-Taani et al. 2022 [21]	385	73.2% good	41.6%	Information access, experience
Lebanon	Hallit et al. 2020 [23]	348	78% accuracy	52%	Economic incentives
Saudi Arabia	Shahid et al. 2017 [19]	412	72% accuracy	45–48%	Urban–rural gap
UAE	Auta et al. 2019 [9]	275	84% accuracy	15%	Strong enforcement
Pakistan	Sakeena et al. 2019 [10]	456	71% accuracy	71%	Limited education
Egypt	Morgan et al. 2011 [22]	320	68% accuracy	71%	Weak enforcement
Australia	Sakeena et al. 2019 [10]	284	86% accuracy	3%	Strict regulation

## Data Availability

The data presented in this study are available upon request from the corresponding author due to privacy and ethical restrictions.
